# Sodium Ferrous Citrate and 5-Aminolevulinic Acid Exert a Therapeutic Effect on Endotoxin-Induced Uveitis in Rats

**DOI:** 10.3390/ijms241713525

**Published:** 2023-08-31

**Authors:** Yuya Otaka, Kazutaka Kanai, Daiki Okada, Noriaki Nagai, Yohei Yamashita, Yoichiro Ichikawa, Kazuki Tajima

**Affiliations:** 1 Department of Small Animal Internal Medicine II, School of Veterinary Medicine, Kitasato University, 35-1 Higashi 23 Ban-Cho, Towada 034-8628, Aomori, Japan; dv20001@st.kitasato-u.ac.jp (Y.O.); dv21002@st.kitasato-u.ac.jp (D.O.); ebisu2101@yahoo.co.jp (Y.Y.); ichikawa-ah@mtj.biglobe.ne.jp (Y.I.); tajima.kazuki@kitasato-u.ac.jp (K.T.); 2 Faculty of Pharmacy, Kindai University, 3-4-1 Kowakae, Higashiosaka 577-8502, Osaka, Japan; nagai_n@phar.kindai.ac.jp

**Keywords:** 5-aminolevulinic acid, endotoxin-induced uveitis, prednisolone, therapeutic effect

## Abstract

The metabolism of 5-aminolevulinic acid (ALA) is more efficient when combined with sodium ferrous citrate (SFC). Our previous study revealed that oral administration of ALA, which has anti-inflammatory properties, and SFC (ALA/SFC) immediately before lipopolysaccharide (LPS) inoculation suppressed endotoxin-induced uveitis (EIU) in rats. However, the therapeutic effect of ALA/SFC post-administration remains unexplored. Hence, this study aimed to evaluate the therapeutic efficacy of ALA/SFC on EIU in rats, which were administered with a gastric gavage of ALA/SFC (100/157 mg/kg) or prednisolone (Pred, 10 mg/kg) after 4 h of LPS inoculation. The treatment groups showed ameliorated clinical scores, inflammatory cells, protein levels in the aqueous humor (AqH), and histopathologic evaluation 24 h after LPS inoculation. Furthermore, the treatment groups had reduced tumor necrosis factor-α, nitric oxide, prostaglandin E_2_, and interleukin-6 levels in the AqH. ALA/SFC demonstrated an anti-inflammatory effect equivalent to that demonstrated by Pred. These findings indicate that ALA/SFC exerts a therapeutic effect on EIU in rats, indicating its clinical usefulness in uveitis treatment.

## 1. Introduction

Uveitis refers to any intraocular inflammation that deteriorates the vision. Significant vision loss has been reported in 20–70% of patients treated for uveitis [1,2,3]. Uveitis can re-occur and cause severe vision loss due to secondary complications such as cataracts, glaucoma, and cystoid macular edema [4]. The pathology is characterized by excessive immune activation, accompanied by elevated inflammatory cytokines, such as tumor necrosis factor (TNF)-α, interleukin (IL)-6, nitric oxide (NO), and prostaglandin E_2_ (PGE_2_) within the aqueous humor (AqH), as presented in both clinical and experimental studies [5,6,7,8].

Corticosteroids are the current mainstays of uveitis treatment to control the inflammatory process [8,9]. Corticosteroids are effective for the acute and chronic treatment of noninfectious eye inflammations but can cause side effects, especially after long-term treatment of chronic and recurrent eye inflammation [10,11,12,13]. Additionally, patients with uveitis having an infectious or corneal ulcer do not benefit from the treatment. Therefore, safe and effective novel therapies are highly desirable.

Endotoxin-induced uveitis (EIU) is induced in rats by injecting lipopolysaccharide (LPS) [14]. Elevated inflammatory cytokines are also observed in EIU, causing cellular infiltration and protein leakage into the AqH 4 h after the LPS injection [5,15,16]. The number of infiltrating cells and protein levels in the AqH peaks 18–24 h after the LPS injection [5,15,16]. The therapeutic efficacy of drugs administered after the onset of inflammation, which is 4–6 h after LPS inoculation, has been investigated in EIU rats to explore their potential as therapeutic agents for uveitis [17,18].

As an amino acid, 5-aminolevulinic acid (ALA) is manufactured by the mitochondria in animals, plants, bacteria, and fungi [19,20]. ALA was discovered in 1953 [21], and its mass production has become recently possible through the application of microbial fermentation technology [22,23]. Protoporphyrin IX (PPIX), which is the precursor of heme, is formed from ALA, and iron ions are incorporated into PPIX to form heme [24]. Interestingly, combining sodium ferrous citrate (SFC) and ALA (ALA/SFC) has demonstrated efficacy in both rats and humans [25,26,27,28]. Many studies have revealed that ALA/SFC has beneficial properties, including antioxidant [29], anti-inflammatory [27], immunomodulatory [30], anticancer [31], and glucose tolerance improvement [28] in diabetes. Additionally, our previous study revealed that ALA/SFC used as a preventive treatment alleviated ocular inflammation in EIU rats [32]. However, its therapeutic potential remained understudied.

This study aimed to investigate the therapeutic efficacy of ALA/SFC in EIU rats and evaluate the anti-inflammatory potential of ALA/SFC compared to prednisolone (Pred).

## 2. Results

### 2.1. Therapeutic Effect of ALA/SFC on Clinical Scoring and the Number of Infiltrating Cells and Protein Levels in AqH

**Figure 1:**
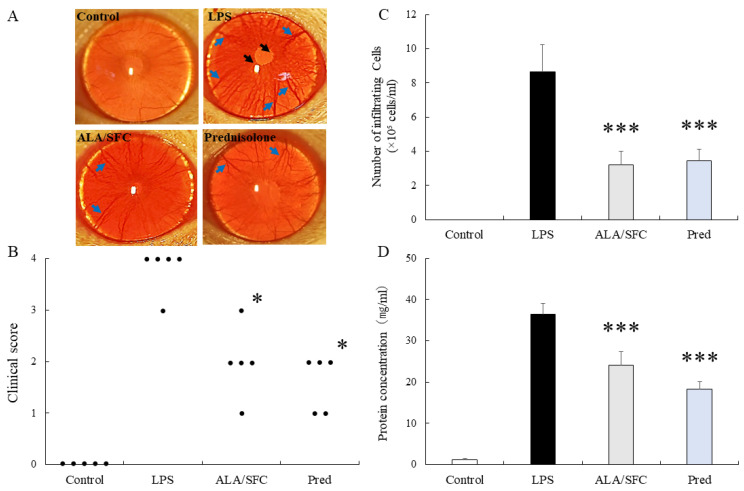
5-Aminolevulinic acid (ALA)/sodium ferrous citrate (SFC) treatment reduced inflammation in endotoxin-induced uveitis (EIU). (**A**) Representative photographs of the eye in each group 24 h after lipopolysaccharide (LPS) injection (black arrow: fibrinous membrane formation; blue arrow: iridial hyperemia). (**B**) The clinical score of EIU was assessed 24 h after the LPS injection. Black dots indicate the clinical score of each rat’s left eye. (**C**,**D**) Effects of ALA/SFC on infiltrating cells and protein levels in the aqueous humor (AqH) 24 h after the LPS injection. Each value represents the mean ± standard deviation (SD) (n = 5). * *p* < 0.05, *** *p* < 0.001, vs. the LPS group. ALA: 5-aminolevulinic acid; AqH: aqueous humor; EIU: endotoxin-induced uveitis; LPS: lipopolysaccharide; Pred: prednisolone; SFC: sodium ferrous citrate.

Clinical scoring in the control group demonstrated no inflammatory response and normal iris vessels. The LPS group demonstrated intense severe iridial hyperemia with anterior chamber flare, fibrinous exudate in the pupillary margin, and a clinical score of 4.0 (median and interquartile range [IQR]; 3.0–4.0, n = 5). The groups treated with ALA/SFC (100/157 mg/kg) and Pred (10 mg/kg) demonstrated mild iridial hyperemia compared with the LPS group, and the clinical scores were significantly reduced to 2.0 (1.5–2.5, *p* < 0.05, vs. LPS) and 2.0 (1.0–2.0, *p* < 0.05, vs. LPS), respectively (Figure 1A,B).

Infiltrating cells were undetected in the AqH in the control group. The number of infiltrating cells was 8.65 ± 1.58 × 10^5^ cells/mL (mean ± standard deviation [SD], n = 5) in the LPS group. The number of infiltrating cells was significantly reduced in the ALA/SFC and Pred groups (ALA/SFC: 3.20 ± 0.79 × 10^5^ cells/mL, *p* < 0.001; Pred at 10 mg/kg: 3.45 ± 0.69 × 10^5^ cells/mL, *p* < 0.001, vs. LPS). The number of infiltrating cells demonstrated no differences between the ALA/SFC and Pred groups (Figure 1C).

The protein levels in the AqH were 1.20 ± 0.28 mg/mL and 36.41 ± 2.71 mg/mL (n = 5) in the control and LPS groups. ALA/SFC and Pred treatment significantly suppressed the protein concentrations in the AqH (ALA/SFC: 24.12 ± 3.3 mg/mL, *p* < 0.001; Pred at 10 mg/kg: 18.31 ± 1.85 mg/mL, *p* < 0.001). ALA/SFC had almost the same effect on the protein levels in the AqH as that for the Pred (Figure 1D).

### 2.2. ALA/SFC Improves Histopathological Evaluation

**Figure 2:**
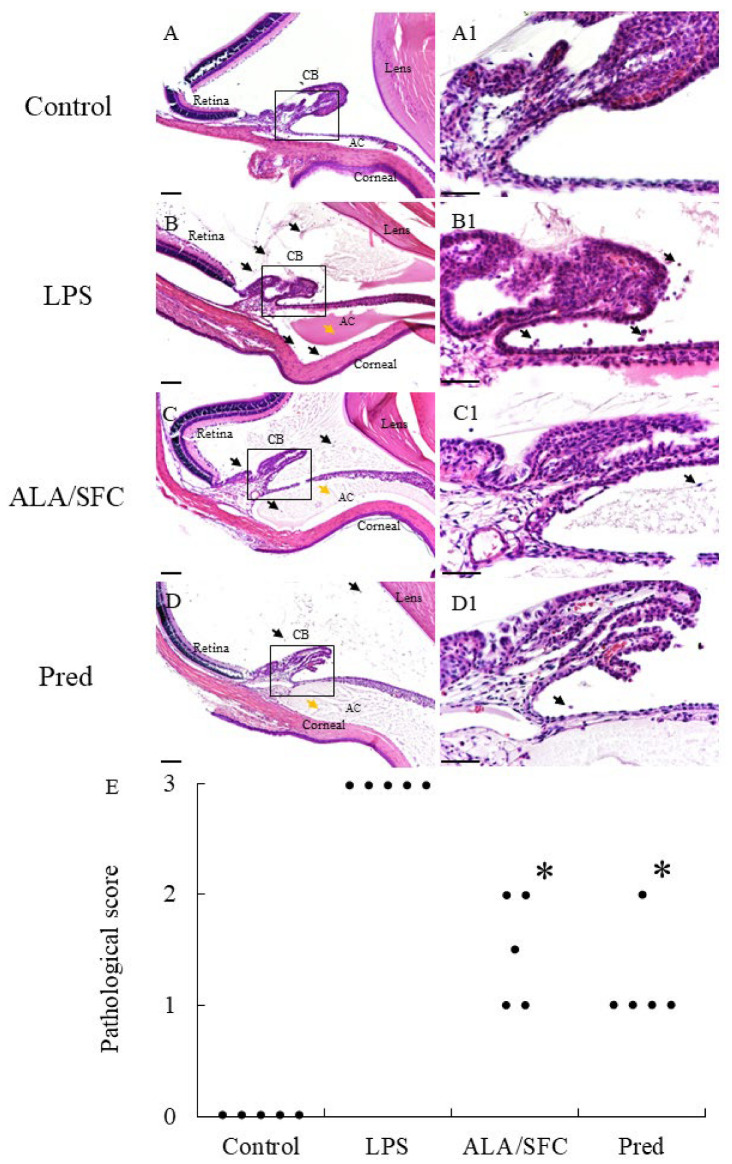
5-Aminolevulinic acid (ALA)/sodium ferrous citrate (SFC) ameliorated the histopathological score of endotoxin-induced uveitis (EIU). (**A**,**A1**) The control group demonstrated no signs of infiltration. (**B**,**B1**) The lipopolysaccharide (LPS) group demonstrated severe inflammatory signs (black arrows: infiltrating cell within the around ciliary body (CB), and anterior chamber (AC); yellow arrows: exudation with dense protein in AC). (**C**,**C1**) ALA/SFC ameliorated inflammatory signs. (**D**,**D1**) Prednisone (Pred) at 10 mg/kg ameliorated inflammatory signs. H&E: hematoxylin and eosin; original magnification, (**A**–**D**): ×100; bars, 100 µm, (**A1**–**D1**): ×400; bars, 50 µm. (**E**) The histopathological score of EIU. Black dots represent the mean histopathological scores of both eyes in each rat (n = 5). * *p* < 0.05, vs. the LPS group. ALA: 5-aminolevulinic acid; AqH: aqueous humor; CB: ciliary body; EIU: endotoxin-induced uveitis; LPS: lipopolysaccharide; Pred: prednisolone; SFC: sodium ferrous citrate.

A histopathological evaluation of the control group revealed normal tissue. The mean histopathological score was 3.0 (3.0–3.0, n = 5) in the LPS group 24 h after the LPS injection, indicating severe uveitis with heavy inflammatory cell infiltration in the iris stroma, ciliary body, and anterior chamber. An improved inflammation response to LPS and a decreased number of infiltration cells resulted in significantly reduced histopathological scores in the ALA/SFC and Pred groups (ALA/SFC: 1.5 [1.0–2.0], *p* < 0.05; Pred at 10 mg/kg: 1.0 [1.0–1.5], *p* < 0.05, vs. LPS). The effect of ALA/SFC on the histopathological scores was almost comparable to Pred (Figure 2).

### 2.3. Therapeutic Effect of ALA/SFC on TNF-α, IL-6, NO, and PGE_2_ Levels in AqH

Increased inflammatory mediators of AqH, such as TNF-α, IL-6, NO, and PGE_2_, are a significant feature in EIU rats. Table 1 shows increases in these inflammatory mediator levels in the LPS group. ALA/SFC and Pred significantly inhibited these inflammatory mediator levels in the AqH (Table 1).

## 3. Discussion

ALA/SFC administration 4 h after the LPS injection significantly reduced the clinical uveitis score, LPS-induced cell infiltrate levels, AqH protein, and inflammatory mediators, and it decreased the histopathological score 24 h after the LPS injection. Moreover, ALA/SFC demonstrated comparable anti-inflammatory effects to Pred at 10 mg.

Numerous investigators have reported that ALA has multiple molecular targets, including transcription factors, cytokines, and apoptosis factors [33,34]. In our previous study, ALA/SFC used as a preventive treatment suppressed inflammatory mediators in EIU rats via inhibition of the NF-κB signaling pathway and activation of the Nrf2/HO-1 signaling pathway [32]. Inflammatory cytokines, such as TNF-α, IL-6, NO, and PGE_2_, which are released by various inflammatory cells, participate in the pathogenesis of EIU [5,6,7,35]. This study revealed that ALA/SFC significantly attenuated ocular inflammation in EIU rats 24 h after the LPS injection. Inflammatory cell infiltration, protein leakage, and TNF-α, IL-6, NO, and PGE_2_ production in the AqH were markedly reduced. Thus, the therapeutic effects of ALA/SFC may also be mediated by inhibiting inflammatory mediators. 

This study used high ALA/SFC doses (100/157 mg/kg). Our previous study with a low ALA/SFC dose (10/15.7 mg/kg) revealed a protective anti-inflammatory effect in EIU rats [32]. However, the dosage used in our previous preliminary studies did not show a therapeutic effect on EIU (Supplementary Table S1). A previous study revealed that administering a high dose of ALA/SFC (100/157 mg/kg) protected against binge alcohol-induced liver injury in HIV transgenic rats [27], and we based our dose of ALA/SFC on this study. The LD_50_ for ALA alone single orally administrated to rats is >2000 mg/kg [36]. The exact toxic dose of ALA/SFC orally administrated to rats is not yet known. However, no side effects or toxicity were observed within 20 h of ALA/SFC oral administration (100/157 mg/kg) in this study. Further studies are essential to evaluate the safety profile and toxicity of ALA/SFC, including its long-term use. As one way to address the issue of toxicity, we intend to elucidate the anti-inflammatory effects of ALA-containing eye drops in order to reduce the potential for systemic side effects.

In summary, to our knowledge, this is the first study to demonstrate the efficacy of ALA/SFC in ocular inflammation after the onset of EIU. Based on results of this study, ALA/SFC might have potential efficacy in treating uveitis.

## 4. Materials and Methods

### 4.1. Reagents

Neopharma Japan (Tokyo, Japan) donated ALA. Komatsuya Corporation (Osaka, Japan) provided SFC. LPS from *Salmonella typhimurium* and Pred were purchased from Sigma (LPS; L6511, Pred; P6004, Sigma-Aldrich, St. Louis, MO, USA).

### 4.2. Animals

This study used 6-week-old male Sprague–Dawley rats, weighing 180–220 g. The rats were purchased from SLC (Hamamatsu, Shizuoka, Japan). They were kept in air-conditioned rooms with a 12 h light/dark cycle. All animal experiments were conducted according to the ARVO statement on the Care and Use of Laboratory Animals. This study was conducted following the Guidelines for Animal Experiments and the Animal Management Manual of the School of Veterinary Medicine, Kitasato University (Approval No. 22-017).

### 4.3. Endotoxin-Induced Uveitis

EIU was induced by subcutaneous injection of LPS at 200 µg diluted in 0.2 mL saline (100 µg each subcutaneously) into each footpad, under anesthesia with isoflurane (Mylan Inc., Canonsburg, PA, USA). A total of 120 rats were randomly divided into four groups, comprising 30 rats in each group: (i) the control group: subcutaneously injected with 0.2 mL of saline and received 10 mL/kg of saline; (ii) the LPS group: subcutaneously injected with 200 µg of LPS and received 10 mL/kg of saline; (iii) the ALA/SFC group: subcutaneously injected with 200 µg of LPS and received 100 mg/kg of ALA and 157 mg/kg of SFC; (iv) the Pred group: subcutaneously injected with 200 µg of LPS and received 10 mg/kg of Pred. Each drug was dissolved in 10 mL/kg of saline 4 h after the LPS injection and administered orally by the gastric tube. The ALA/SFC dosage ratio (molar ratio; ALA: SFC = 1:0.5) was determined based on previous studies [27,32]. Clinical scoring was assessed 24 h after the LPS injection, and AqH and eye samples were collected and analyzed after euthanasia with isoflurane in deep anesthesia.

### 4.4. Evaluation of Clinical Scoring

The rats were evaluated for clinical scoring before euthanasia 24 h after the LPS injection. The clinical scores were assessed from scores 0–4, following a previously published system [32]. Two observers who were not informed about the drug treatment assessed clinical scores of the left eye. Images of clinical symptoms were recorded with a digital camera (^®^PENTAX Q-S1, RICOH Imaging, Co, Ltd., Tokyo, Japan) (n = 5 in each group).

### 4.5. Infiltrating Cell Count and Protein Level Measurement in AqH

This analysis was performed as previously described [32]. The rats were euthanized via overdose with isoflurane 24 h after the LPS injection. AqH in both eyes was obtained by a 30-gauge needle under a microscope (MEGA-21; Inami and Co., Ltd., Tokyo, Japan). AqH from the control group was not diluted for cell counting, but AqH from other groups was diluted 10-fold with sterile saline. The AqH samples were diluted 2-fold with Türk-staining solution and counted using a hematocytometer. Next, the AqH samples were centrifuged (2500 rpm, 5 min, 4 °C). The total protein concentration in the supernatant in AqH was determined using a bicinchoninic acid protein assay kit (Pierce, Rockford, IL, USA) and diluted 5-fold with sterile saline for the control group and 100-fold for the other groups (n = 5 in each group).

### 4.6. Evaluation of Histopathologic Scores

The rats were euthanized 24 h after the LPS injection, and both eyes were promptly enucleated, stored in 4% paraformaldehyde in phosphate-buffered saline for 24 h at 4 °C, dehydrated in a graded ethanol series, and embedded in paraffin. Sagittal sections of 3 µm were cut near the optic disc and stained with hematoxylin and eosin (H&E). The histopathologic scores were graded 0–3, as previously described [32]. A histopathological evaluation was performed on both eyes of 5 rats in each group, and the evaluators were unaware of the drug treatment. Each rat obtained a mean score for both eyes (n = 5 in each group).

### 4.7. Measurement of Cytokine Levels in AqH

The TNF-α, IL-6, and PGE_2_ levels in the AqH were measured by enzyme-linked immunosorbent assay (TNF-α: KE20001; Proteintech Group Inc., Rosemont, IL, USA; IL-6: #BMS625; Thermo Fisher Scientific, Waltham, MA, USA; PGE_2_: 500141; Cayman Chemical Co., Ann Arbor, MI, USA) following the manufacturer’s instructions. The nitrate/nitrite levels in the AqH were measured by NO_2_/NO_3_ colorimetric assay kit (NK05; Dojindo Molecular Technologies Inc., Kumamoto, Japan) (n = 5 in each group).

### 4.8. Statistical Analysis

Nonparametric data, clinical and histopathologic scores, are expressed as median (IQR). Parametric data are expressed as mean ± SD. Statistical analyses were performed using EZR version 1.55, which is an open-source software [37]. One-way analysis of variance and Tukey’s post-hoc multiple comparison tests were used to analyze significant differences among groups in cell, protein, and cytokine levels in AqH. The Kruskal–Wallis and Steel–Dwass post-hoc multiple comparison tests were conducted to analyze significant differences in clinical and histopathologic scores between the two treatment groups. The significance was marked with *p*-values of <0.05 for all analyses.

## Figures and Tables

**Table 1:** 5-Aminolevulinic acid (ALA)/sodium ferrous citrate (SFC) suppressed the levels of inflammatory mediators in the aqueous humor (AqH) of endotoxin-induced uveitis (EIU).

Group	TNF-α (pg/mL)	IL-6 (pg/mL)	NO (µM)	PGE_2_ (pg/mL)
Control	8.53 ± 5.29	16.79 ± 11.91	1.98 ± 4.43	197.33 ± 40.09
LPS	64.95 ± 9.09	86.28 ± 37.84	199.12 ± 35.31	1947.40 ± 219.27
ALA/SFC	23.27 ± 14.41 ^c^	24.67 ± 18.32 ^a^	149.21 ± 30.22 ^a^	1137.17 ± 100.40 ^c^
Pred	26.26 ± 9.62 ^c^	28.70 ± 25.18 ^a^	124.19 ± 12.00 ^b^	1021.47 ± 113.21 ^c^

Each value represents the mean ± SD (n = 5). ^a^ *p* < 0.05, ^b^ *p* <0.01, ^c^ *p* < 0.001, compared with the LPS group. ALA: 5-Aminolevulinic acid; AqH: aqueous humor; EIU: endotoxin-induced uveitis; IL: interleukin; LPS: lipopolysaccharide; NO: nitric oxide; PGE_2_: prostaglandin E_2_; Pred: prednisolone; SFC: sodium ferrous citrate; TNF-α: tumor necrosis factor.

## Data Availability

The data presented in this study are available on request from the corresponding author.

## Supplementary Materials

The following supporting information can be downloaded at: https://www.mdpi.com/article/10.3390/ijms241713525/s1.
