# Supplementary material for: Sodium Ferrous Citrate and 5-Aminolevulinic Acid Exert a Therapeutic Effect on Endotoxin-Induced Uveitis in Rats
Source: Int J Mol Sci. 2023 Aug 31;24(17):13525. doi: 10.3390/ijms241713525 (PMC10487957; doi:10.3390/ijms241713525)
Supplement: Supplementary file 1 [file ijms-24-13525-s001.zip › ijms-2543555-supplementary.pdf]

Supplementary Table S1. Effect of a low dose of 5-Aminolevulinic acid (ALA)/sodium ferrous citrate (SFC) on endotoxin-induced uveitis (EIU)

Group	Clinical scores	Number of infiltrating cells in AqH ($\times 10^5$ cells/ml^{-1})	Protein concentration in AqH (mg/ml^{-1})
ALA/SFC (10/15.7 mg/kg^{-1})	2.8 ± 0.45	8.1 ± 2.66	29.44 ± 6.36

Each value represents the mean ± SD (n = 5). ALA: 5-Aminolevulinic acid; AqH: aqueous humor; EIU: endotoxin-induced uveitis; LPS: lipopolysaccharide; Pred: prednisolone; SFC: sodium ferrous citrate.
